# Phase-specific effects of guar meal and β-mannanase on growth performance and nutrient digestibility in broilers

**DOI:** 10.1007/s11250-026-05275-4

**Published:** 2026-08-10

**Authors:** Haroon Ur Rashid, Taimoor Khan, Muhammad Tahir, Muhammad Daud, Damiano Cavallini, Giovanni Buonaiuto

**Affiliations:** 1 Livestock and Dairy Development Department, Khyber Pakhtunkhwa, Pakistan; 2 Livestock Research and Development Station and Animal Analytical Laboratory Dir lower, Khyber Pakhtunkhwa, Pakistan; 3 Ayubi Model College, Near Scout Chawni Balambat, Timergara, Pakistan; 4 Department of Animal Nutrition, University of Agriculture Peshawar, Peshawar, Pakistan; 5 Department of Veterinary Medical Sciences, University of Bologna, Bologna, Italy; 6 Department of Veterinary Sciences, University of Parma, Parma, Italy

**Keywords:** Guar meal, β-mannanase, Broiler chickens, Nutrient digestibility, Feed efficiency

## Abstract

This study evaluated the phase-specific effects of dietary guar meal (GM) inclusion, with or without β-mannanase supplementation, on growth performance, nutrient digestibility, and energy utilization in broiler chickens. A total of 300 male Ross 308 broilers were assigned to eight experimental diets in a 4 × 2 factorial arrangement, consisting of four GM inclusion levels (0%, 4%, 8%, and 12%) with or without β-mannanase supplementation (0.35 g/kg), and were evaluated during the starter (7–21 d) and grower (22–35 d) phases. During the starter phase, broilers fed 4% GM supplemented with β-mannanase achieved growth performance comparable to the control diet, whereas increasing GM inclusion significantly reduced body weight gain, crude protein digestibility, and apparent metabolizable energy (AME; *P* < 0.05). β-mannanase supplementation partially alleviated these negative effects and fully restored crude protein digestibility at 4% GM. During the grower phase, broilers tolerated up to 8% GM without major reductions in growth performance, whereas 12% GM consistently impaired productive responses. Enzyme supplementation further enhanced body weight gain and protein digestibility across GM inclusion levels; however, its efficacy declined at 12% GM, where performance and energy utilization remained significantly lower than the control diet (*P* < 0.05). These findings demonstrate that broiler responses to GM are strongly age-dependent and identify phase-specific tolerance thresholds for dietary inclusion. Under the conditions of this study, 4% GM during the starter phase and up to 8% GM during the grower phase can be successfully incorporated when supported by β-mannanase supplementation. This strategy may facilitate the sustainable utilization of guar meal as an agro-industrial by-product while maintaining productive performance and nutrient efficiency in broiler production systems.

## Introduction

The growing demand for animal protein, together with increasing pressure on natural resources and feed costs, poses significant challenges to the sustainability of modern poultry production systems (El-Sabrout et al. 2026). Broiler production has expanded considerably over recent decades and represents one of the most important source of high-quality animal protein worldwide (Bist et al. 2024). However, the poultry industry continues to face the dual challenge of maintaining high productive performance while ensuring economic sustainability (Nassar 2026). As a result, considerable attention has been directed toward identifying alternative protein sources that can partially replace conventional feed ingredients. Nevertheless, many unconventional feedstuffs contain anti-nutritional factors (ANFs) that may impair nutrient utilization and animal performance (Vlaicu et al. 2024).

Guar (*Cyamopsis tetragonoloba*) is a drought-resistant legume mainly cultivated in India and Pakistan (Kuravadi et al. 2013). Although its seeds are primarily processed for galactomannan gum extraction (Mudgil et al. 2014), the residual by-product, GM, contains approximately 35 to 47.5% crude protein and has a favorable amino acid profile, making it a potentially valuable protein source for poultry diets (Saeed et al. 2017; Biel and Jaroszewska 2019). The use of GM may also contribute to the sustainable utilization of agro-industrial by-products by reducing dependence on conventional protein sources and promoting circular economy practices (Hickmann et al. 2021). However, its application in broiler nutrition remains limited because high dietary inclusion levels have frequently been associated with reduced growth performance, poorer feed efficiency, and increased mortality (Chuppava et al. 2022). These adverse effects are mainly attributed to residual guar gum and ANFs, particularly β-mannans, which increase intestinal viscosity and negatively affect nutrient digestion and absorption (Lee et al. 2003a, b).

Several studies have shown that higher inclusion rates (7.5–18%) of guar meal negatively affect broiler performance, largely due to increased digesta viscosity caused by residual guar gum (Mallela et al. 2019; Milczarek et al. 2022). In contrast, lower inclusion levels (2.5–5%) may not compromise performance and can even support growth when used to partially replace soybean meal (Daskiran et al. 2004). Mishra et al. (2013) found that gradually increasing guar meal inclusion (2% in the starter phase and 5% in grower and finisher phases) did not adversely affect weight gain, feed conversion ratio, or carcass quality. Similarly, Gheisari et al. (2011) and Mallela et al. (2019) reported improvements in productivity and carcass parameters at lower inclusion levels (3–9%), with performance deterioration occurring at levels above 12%.

To overcome the limitations associated with β-mannans, β-mannanase supplementation has emerged as a promising nutritional strategy (Saeed et al. 2019; Jang et al. 2024). This enzyme, hydrolyzes β-mannans in the gastrointestinal tract, thereby reducing digesta viscosity and improving nutrient digestibility (Saeed et al. 2019). Positive effects of β-mannanase supplementation on growth performance, feed efficiency, and nutrient utilization have been reported in several monogastric species, including broiler chickens (Kiarie et al. 2021; Carvalho et al. 2023; Genova et al. 2023).

Despite the growing interest in guar meal as an alternative protein source, no previous study has systematically evaluated the interaction between dietary GM inclusion level, β-mannanase supplementation, and growth phase in broiler chickens. Therefore, the present study aimed to investigate the phase-specific effects of increasing dietary GM levels, with or without β-mannanase supplementation, on growth performance, crude protein digestibility, and energy utilization in broilers. The study was designed to identify inclusion thresholds that maximize nutrient efficiency and productive performance while supporting the sustainable use of agro-industrial by-products in poultry nutrition.

## Materials and methods

### Ethic statement

The present experiment was approved by the Ethical Committee of the University of Peshawar (ID: 2024/2025). The trial was conducted at the Center of Animal Nutrition, Directorate of Livestock Research and Development, Peshawar, under the supervision of the Department of Animal Nutrition.

### Birds, housing, and management

A total of 300 one-day-old male Ross 308 chicks were obtained from a commercial hatchery. All chicks, originating from the same breeder flock and hatching batch, were vaccinated at the hatchery against infectious bronchitis, Newcastle disease, and infectious bursal disease. Chicks were initially reared under uniform management conditions in an open-sided, naturally ventilated poultry house with sawdust litter.

On day 7, birds were individually weighed, and 240 healthy chicks with homogeneous body weight were selected and transferred to an environmentally controlled experimental facility. Birds were randomly assigned to experimental treatments. Each pen had a concrete floor covered with sawdust litter (3–4 kg/m²), a circular pan feeder (2 cm feeder space/bird), and five nipple drinkers (5 birds/nipple). A lighting schedule of 23 h light and 1 h dark was applied from day 0 to 7, followed by 18 h light and 6 h dark from day 8 to 35. Ambient temperature was maintained at 32–34 °C during the first week and gradually reduced by approximately 2–3 °C per week until reaching 22–24 °C by the end of the trial. Feed and water were provided ad libitum throughout the study. Experimental diets were offered from day 7 to 35 and divided into two feeding phases: starter (day 7–21) and grower (day 22–35).

### Experimental design and diet formulation

The GM was procured from a commercial supplier (Classic Feeds, Mansabdar, Swabi) and analyzed for proximate composition at the University of Agriculture, Peshawar. Eight experimental diets were formulated in a 4 × 2 factorial arrangement: four GM inclusion levels (0%, 4%, 8%, and 12%), each with or without β-mannanase supplementation (0.35 g/kg). All diets were iso-nitrogenous and iso-caloric, formulated according to Aviagen (2019) and NRC (1994) guidelines.

Energy adjustments were achieved by varying vegetable oil content, and essential amino acids (lysine, methionine, threonine, and total sulfur amino acids) were added as needed. All feed ingredients were sourced from the same feed mill and analyzed for nutrient consistency. Ingredient composition and nutrient values are reported in Tables 1 and 2.

**Table 1:** Ingredients used in formulation^1^

Ingredients	Starter				Grower			
	GM_0_	GM_4_	GM_8_	GM_12_	GM_0_	GM_4_	GM_8_	GM_12_
Corn, grain	55.907	55.246	55.046	54.781	62.563	61.567	61.349	60.186
Soybean Meal-44%	22.500	19.118	16.013	11.768	16.890	13.925	10.974	7.769
Gluten Meal-60	5.000	5.000	4.244	5.000	5.000	4.683	4.970	4.340
Canola Meal	5.000	5.000	4.083	4.000	5.000	5.000	3.000	3.500
Guar meal	0.000	4.000	8.000	12.000	0.000	4.000	8.000	12.000
Vegetable Oil	2.841	2.974	3.000	3.000	2.751	2.991	3.000	3.300
Poultry by-product Meal	3.000	3.000	4.000	3.700	3.000	3.000	3.300	3.500
Sunflower Meal	2.161	2.000	2.000	2.000	2.000	2.000	2.500	2.500
Limestone	1.300	1.327	1.335	1.372	1.058	1.084	1.121	1.136
Dicalcium Phosphate	1.397	1.365	1.252	1.255	0.963	0.926	0.885	0.827
Salt	0.425	0.426	0.418	0.422	0.400	0.400	0.400	0.400
DL-Methionine	0.185	0.198	0.212	0.230	0.098	0.112	0.123	0.137
L-Lysine HCL	0.150	0.195	0.232	0.330	0.156	0.191	0.259	0.284
Threonine	0.013	0.030	0.044	0.100	0.000	0.000	0.000	0.000
Vit min premix	0.120	0.120	0.120	0.120	0.120	0.120	0.120	0.120

^1^GM_0_ = basal diet; GM_4_ = diet containing 4% guar meal (GM); GM_8_ = 8% GM; GM_12_ = 12% GM

**Table 2:** Chemical composition of experimental diets^1^

	Starter				Grower			
	GM_0_	GM_4_	GM_8_	GM_12_	GM_0_	GM_4_	GM_8_	GM_12_
AME^2^, Kcal/kg	3070	3070	3070	3070	3120	3120	3120	3120
Protein, %	23.0	23.0	23.0	23.0	20.9	20.9	20.9	20.9
Lysine, %	1.28	1.27	1.28	1.28	1.00	1.00	1.00	1.00
Methionine + Cystine, %	0.90	0.90	0.90	0.90	0.86	0.86	0.86	0.86
Methionine, %	0.58	0.58	0.57	0.58	0.61	0.61	0.61	0.61
Threonine, %	0.88	0.88	0.87	0.88	0.82	0.82	0.82	0.82
Tryptophan, %	0.31	0.31	0.31	0.31	0.31	0.31	0.31	0.31
Calcium, %	1.00	1.00	1.00	1.00	0.90	0.90	0.90	0.90
Non phytate P, %	0.45	0.45	0.45	0.45	0.43	0.43	0.43	0.43

^1^GM_0_ = basal diet; GM_4_ = diet containing 4% guar meal (GM); GM_8_ = 8% GM; GM_12_ = 12% GM

^2^Apparent Metabolizable Energy

On day 7, 240 chicks were randomly allocated to eight dietary treatments in a completely randomized design, with three replicate pens per treatment and ten birds per pen. The treatment groups were: GM_0_ (basal diet), GM_0E_ (basal + enzyme), GM_4_ (4% GM), GM_4E_ (4% GM + enzyme), GM_8_ (8% GM), GM_8E_ (8% GM + enzyme), GM_12_ (12% GM), and GM_12E_ (12% GM + enzyme). Feed and water were provided *ad libitum* throughout the trial.

### Measuring parameters and data collection

All birds were weighed individually at placement (day 7), and subsequently on days 14, 21, 28, and 35. Weekly feed intake (FI) was calculated on a pen basis by subtracting leftover feed from the total offered. Mortality was recorded daily, and the weight of deceased birds was used to adjust performance parameters. Body weight (BW), daily weight gain (DWG), and feed conversion ratio (FCR) were calculated per phase and for the entire study period.

AME was determined using the total excreta collection method according to Dourado et al. (2010). On days 19 and 33, four birds representative of the average pen body weight were randomly selected from each replicate and individually housed in metabolic cages. Following a one-day adaptation period, total fecal collection was conducted for 48 h during both the starter and grower phases. Excreta were collected twice daily (morning and evening), and only uncontaminated samples were retained for analysis.

Feed and fecal samples were oven-dried at 105 °C to determine dry matter (DM) content, according to AOAC (2005) 934.01 method. Ash content was determined by incineration of the dried samples in a muffle furnace at 550 °C for 8 h, following AOAC (2005) method 942.05. Organic matter (OM) was calculated by difference as 100 − ash content. CP was determined by the Kjeldahl method, including digestion, distillation, and titration, according to AOAC (2005) method 2001.11. Total nitrogen content was calculated from titration results and converted to crude protein using the standard factor of 6.25. All chemical analyses were expressed on a dry matter basis.

### Statistical analysis

Data were analyzed using the General Linear Model (GLM) procedure of SAS (Version 9.4; SAS Institute Inc., Cary, NC, USA). A two-way ANOVA was performed according to a 4 × 2 factorial arrangements to evaluate the effects of dietary guar meal (GM) inclusion level (0, 4, 8, and 12%), β-mannanase supplementation (0–0.35 g/kg diet), and their interaction.

The statistical model used was:

$$\eqalign{ {{\rm{Y}}_{{\rm{ijk}}}}& {\rm{ = \mu + G}}{{\rm{M}}_{\rm{i}}}{\rm{ + Enzym}}{{\rm{e}}_{\rm{j}}} \cr & {\rm{ + }}{\left( {{\rm{GM \times Enzyme}}} \right)_{{\rm{ij}}}}{\rm{ + }}{{\rm{\varepsilon }}_{{\rm{ijk}}}} \cr}$$

where Y_ijk_ is the observed variable, µ is the overall mean, GM_i_ is the fixed effect of guar meal inclusion level, Enzyme_j_ is the fixed effect of β-mannanase supplementation, (GM × Enzyme)_ij_ is the interaction effect, and ε_ijk_ is the residual error. When significant effects were detected, means were separated using Duncan’s Multiple Range Test. Statistical significance was declared at *P* ≤ 0.05.

## Results

### Growth performance and nutrient utilization during the starter phase

Across the starter phase (day 7–21), broiler performance was significantly affected by dietary GM inclusion level and β-mannanase supplementation (Table 3). Birds fed the control diet (GM0) and those receiving 4% GM supplemented with β-mannanase (GM4E) achieved comparable body weight gain (BWG). In contrast, BWG decreased significantly as dietary GM inclusion increased, with the lowest values observed in birds fed 12% GM without β-mannanase supplementation (*P* < 0.05). β-mannanase supplementation partially alleviated this reduction at the highest inclusion level, although performance did not fully recover to control values.

**Table 3:** The effects of guar meal (GM) enriched with and without b-mannannase on the performance of broiler chicks during the first two weeks of experiment

Diets^1^	Body weight gain	Feed intake	Feed conversion ratio
1st week (d 7–14)			
GM_0_	170.93^ab^ ± 10.77	260.13 ± 22.20	1.52 ± 0.06
GM_0E_	178.03^a^ ± 12.51	273.13 ± 14.02	1.53 ± 0.05
GM_4_	169.37^ab^ ± 10.36	276.57 ± 19.29	1.64 ± 0.18
GM_4E_	170.87^ab^ ± 7.96	272.20 ± 10.26	1.60 ± 0.10
GM_8_	161.37^ab^ ± 15.92	276.10 ± 3.29	1.72 ± 0.16
GM_8E_	166.17^ab^ ± 10.77	289.73 ± 16.76	1.74 ± 0.02
GM_12_	152.90^b^ ± 11.38	267.97 ± 15.14	1.76 ± 0.20
GM_12E_	160.10^ab^ ± 14.65	265.93 ± 19.62	1.67 ± 0.11
2nd week (d 15–21)			
GM_0_	440.83^a^ ± 11.82	935.67^a^ ± 15.18	2.12^ab^ ± 0.04
GM_0E_	437.83^a^ ± 13.58	899.83^ab^ ± 25.76	2.06^b^ ± 0.07
GM_4_	422.13^ab^ ± 9.57	850.93^c^ ± 22.91	2.02^b^ ± 0.04
GM_4E_	431.83^a^ ± 6.22	912.53^ab^ ± 21.47	2.11^ab^ ± 0.03
GM_8_	419.53^ab^ ± 9.57	900.17^ab^ ± 20.77	2.14^ab^ ± 0.09
GM_8E_	430.57^a^ ± 5.50	928.30^ab^ ± 14.04	2.16^ab^ ± 0.02
GM_12_	399.57^b^ ± 21.69	892.10^b^ ± 13.19	2.24^a^ ± 0.15
GM_12E_	421.70^ab^ ± 16.21	906.10^ab^ ± 16.11	2.15^ab^ ± 0.10

Means with different superscripts within a column differ significantly (*P* < 0.05)

^1^GM_0_ = basal diet; GM_0E_ = basal diet + β-mannanase; GM_4_ = diet containing 4% GM; GM_4E_ = 4% GM + β-mannanase; GM_8_ = 8% GM; GM_8E_ = 8% GM + β-mannanase; GM_12_ = 12% GM; GM_12E_ = 12% GM + β-mannanase

Feed intake was affected by dietary treatment, with the highest values observed in GM8E and the lowest in GM4 (*P* < 0.05). The FCR deteriorated with increasing GM inclusion, reaching the highest values in GM_12_. Enzyme supplementation improved FCR at all GM levels, resulting in values comparable to the control diet at 4% GM, while only partial improvements were observed at 8% and 12% inclusion.

Weekly performance data (weeks 1 and 2) were consistent with the aggregated starter phase results (Table 4), confirming a stable response pattern over time. In particular, the negative effects of higher GM inclusion became more evident during the second week, whereas enzyme supplementation exerted a consistent mitigating effect across both weeks. The CP digestibility decreased significantly as dietary GM inclusion increased (*P* < 0.05; Table 5). The lowest CP digestibility was observed in birds fed 12% GM without enzyme (*P* < 0.05). β-mannanase supplementation significantly improved CP digestibility at all GM levels, fully restoring values to those of the control diet at 4% GM and partially mitigating the decline at 8% and 12% inclusion.

**Table 4:** The effects of guar meal (GM) enriched with and without b-mannannase on the performance of broiler chicks during the starter phase of experiment

Diets^1^	Body weight gain	Feed intake	Feed conversion ratio
GM_0_	611.77^a^ ± 16.72	1195.80^ab^ ± 30.87	1.95^bc^ ± 0.04
GM_0E_	615.87^a^ ± 12.23	1172.97^abc^ ± 31.16	1.90^c^ ± 0.03
GM_4_	591.50^ab^ ± 14.17	1127.50^c^ ± 32.74	1.91^c^ ± 0.03
GM_4E_	602.70^ab^ ± 13.82	1184.73^ab^ ± 15.99	1.97^bc^ ± 0.02
GM_8_	580.90^b^ ± 13.76	1176.27^abc^ ± 18.75	2.03^ab^ ± 0.08
GM_8E_	596.73^ab^ ± 16.12	1218.03^a^ ± 28.03	2.04^ab^ ± 0.03
GM_12_	552.47^c^ ± 10.48	1160.07^bc^ ± 20.36	2.10^a^ ± 0.06
GM_12E_	581.80^b^ ±14.66	1172.03^abc^ ± 26.38	2.01^ab^ ± 0.09

Means with different superscripts within a column differ significantly (*P* < 0.05)

^1^GM_0_ = basal diet; GM_0E_ = basal diet + β-mannanase; GM_4_ = diet containing 4% GM; GM_4E_ = 4% GM + β-mannanase; GM_8_ = 8% GM; GM_8E_ = 8% GM + β-mannanase; GM_12_ = 12% GM; GM_12E_ = 12% GM + β-mannanase

**Table 5:** The effect of guar meal (GM) enriched with and without b-mannannase on the crude protein (CP) digestibility and apparent metabolizable energy (AME) in the starter phase of broiler chicks

Diets^1^	CP (%)	AME (kcal/kg)
GM_0_	70.51^ab^ ± 0.26	3040.81^a^ ± 25.55
GM_0E_	71.16^a^ ± 0.21	3052.76^a^ ± 23.85
GM_4_	69.92^b^ ± 0.17	2978.96^b^ ± 28.46
GM_4E_	70.41^ab^ ± 0.20	2985.50^b^ ± 20.48
GM_8_	68.02^d^ ± 0.17	2858.97^c^ ± 28.24
GM_8E_	69.01^c^ ± 0.11	2883.26^c^ ± 23.57
GM_12_	66.13^e^ ± 0.12	2757.71^e^ ± 22.11
GM_12E_	67.81^d^ ± 0.11	2805.41^d^ ± 24.42

Means with different superscripts within a column differ significantly (*P* < 0.05)

^1^GM_0_ = basal diet; GM_0E_ = basal diet + β-mannanase; GM_4_ = diet containing 4% GM; GM_4E_ = 4% GM + β-mannanase; GM_8_ = 8% GM; GM_8E_ = 8% GM + β-mannanase; GM_12_ = 12% GM; GM_12E_ = 12% GM + β-mannanase

The AME was negatively affected by increasing GM inclusion, with stable values observed up to 4% GM and a marked reduction at 12% GM. β-mannanase supplementation resulted in modest improvements in AME; however, values at the highest GM level remained significantly lower than those of the control diet, indicating limited recovery of energy utilization under high GM inclusion.

### Growth performance and nutrient utilization during the grower phase

During the grower phase (day 22–35), broiler responses to GM inclusion differed from those observed during the starter phase (Tables 6 and 7). Birds fed 8% GM maintained BWG comparable to those receiving 4% GM, whereas birds fed 12% GM showed lower BWG. Nevertheless, BWG remained significantly reduced at 12% GM, regardless of enzyme supplementation (Table 7). β-mannanase supplementation improved BWG across all GM levels, with birds fed GM_4E_ and GM_8E_ achieving performance numerically higher than the control diet (Table 7). FI was not significantly affected by treatment (*P* > 0.05), although numerically greater values were observed in birds fed GM8E. Despite the numerically greater FI observed in GM8E, FCR remained numerically higher than that observed in GM4E (Table 7).

**Table 6:** The effects of guar meal (GM) enriched with and without b-mannannase on the performance of broiler chicks during the seconds two weeks of experiment

Diets^1^	Body weight gain	Feed intake	Feed conversion ratio
3rd week (d 22–28)			
GM_0_	421.63^ab^ ± 13.34	901.97^bc^ ± 20.34	2.14^ab^ ± 0.10
GM_0E_	439.83^a^ ± 22.07	939.60^ab^ ± 27.56	2.14^ab^ ± 0.06
GM_4_	416.87^ab^ ± 15.81	816.33^d^ ± 23.50	1.96^c^ ± 0.06
GM_4E_	432.27^a^ ± 19.87	880.70^c^ ± 17.06	2.04^bc^ ± 0.09
GM_8_	408.50^ab^ ± 10.31	872.57^c^ ± 23.26	2.14^ab^ ± 0.09
GM_8E_	436.73^a^ ± 18.05	973.83^a^ ± 14.41	2.23^a^ ± 0.12
GM_12_	396.40^b^ ± 14.61	812.73^d^ ± 26.49	2.05^bc^ ±0.09
GM_12E_	419.07^ab^± 13.14	910.80^bc^ ± 22.22	2.17^ab^ ± 0.03
4th week (d 29–35)			
GM_0_	411.10^a^ ± 12.04	885.97^a^ ± 16.03	2.11^ab^ ± 0.04
GM_0E_	422.50^a^ ± 11.91	865.40^a^ ± 14.30	2.05^ab^ ± 0.10
GM_4_	405.40^ab^ ± 13.72	825.20^b^ ± 24.73	2.04^ab^ ± 0.13
GM_4E_	418.07^a^ ± 12.91	825.37^b^ ± 21.10	1.98^b^ ± 0.10
GM_8_	403.67^ab^ ± 12.41	880.40^a^ ± 5.15	2.18^a^ ± 0.06
GM_8E_	417.03^a^ ± 14.04	885.97^a^ ± 29.94	2.13^ab^ ± 0.13
GM_12_	382.73^b^ ± 11.89	801.13^b^ ± 22.10	2.09^ab^ ± 0.05
GM_12E_	401.27^ab^ ± 10.71	808.70^b^ ± 17.67	2.02^ab^ ± 0.10

Means with different superscripts within a column differ significantly (*P* < 0.05)

^1^GM_0_ = basal diet; GM_0E_ = basal diet + β-mannanase; GM_4_ = diet containing 4% GM; GM_4E_ = 4% GM + β-mannanase; GM_8_ = 8% GM; GM_8E_ = 8% GM + β-mannanase; GM_12_ = 12% GM; GM_12E_ = 12% GM + β-mannanase

**Table 7:** The effects of guar meal (GM) enriched with and without b-mannannase on the performance of broiler chicks during the grower phase of experiment

Diets^1^	Body weight gain	Feed intake	Feed conversion ratio
GM_0_	832.73^abc^ ± 19.85	1771.67^bc^ ± 28.32	2.13^ab^ ± 0.04
GM_0E_	862.33^a^ ± 23.25	1805.00^b^ ± 29.00	2.09^abc^ ± 0.04
GM_4_	822.27^bc^ ± 13.35	1641.53^f^ ± 27.12	1.99^d^ ± 0.04
GM_4E_	850.33^abc^ ± 24.95	1706.07^e^ ± 28.35	2.01^cd^ ± 0.09
GM_8_	812.17^cd^ ± 18.39	1752.97^cd^ ± 27.62	2.16^ab^ ± 0.05
GM_8E_	853.77^ab^ ± 11.63	1859.80^a^ ± 16.67	2.18^a^ ± 0.01
GM_12_	779.13^d^ ± 25.88	1613.87^f^ ± 29.43	2.07^bcd^ ± 0.04
GM_12E_	820.33^bc^ ± 23.80	1719.50^de^ ± 6.95	2.10^abc^ ± 0.06

Means with different superscripts within a column differ significantly (*P* < 0.05)

^1^GM_0_ = basal diet; GM_0E_ = basal diet + β-mannanase; GM_4_ = diet containing 4% GM; GM_4E_ = 4% GM + β-mannanase; GM_8_ = 8% GM; GM_8E_ = 8% GM + β-mannanase; GM_12_ = 12% GM; GM_12E_ = 12% GM + β-mannanase

Weekly data from the third and fourth weeks (Table 6) followed similar trends, confirming the consistency of the grower-phase response. β-mannanase supplementation consistently enhanced BWG and moderated FCR, although the magnitude of the response was lower at the highest GM inclusion level. CP digestibility during the grower phase decreased progressively with increasing GM inclusion, with the lowest values recorded in birds fed 12% GM (Table 8). β-mannanase supplementation significantly improved CP digestibility at all GM levels, with values at 4% GM comparable to the control diet and partial recovery observed at 8% and 12% GM.

**Table 8:** The effect of guar meal (GM) enriched with and without b-mannannase on the crude protein (CP) digestibility and apparent metabolizable energy (AME) in the grower phase of broiler chicks

Diets^1^	CP (%)	AME (kcal/kg)
GM_0_	74.41^a^ ± 0.27	3096.93^a^ ± 24.45
GM_0E_	74.41^a^ ± 0.18	3127.27^a^ ± 23.88
GM_4_	73.30^c^ ± 0.26	3086.65^a^ ± 25.15
GM_4E_	73.71^bc^ ± 0.24	3103.31^a^ ± 22.89
GM_8_	71.87^e^ ± 0.28	2906.44^bc^ ± 20.78
GM_8E_	72.66^d^ ± 0.25	2941.16^b^ ± 19.81
GM_12_	67.09^g^ ± 0.22	2835.62^d^ ± 20.19
GM_12E_	69.20^f^ ± 0.23	2874.51^c^ ± 21.55

Means with different superscripts within a column differ significantly (*P* < 0.05)

^1^GM_0_ = basal diet; GM_0E_ = basal diet + β-mannanase; GM_4_ = diet containing 4% GM; GM_4E_ = 4% GM + β-mannanase; GM_8_ = 8% GM; GM_8E_ = 8% GM + β-mannanase; GM_12_ = 12% GM; GM_12E_ = 12% GM + β-mannanase

AME was highest in birds fed enzyme-supplemented diets with 0% and 4% GM and declined significantly at 8% and 12% GM (Table 8). Although enzyme supplementation improved AME at all inclusion levels, values at 12% GM remained significantly lower than those of the control diet, confirming a limited capacity to restore energy utilization under high GM inclusion.

## Discussion

The present study evaluated the phase-specific effects of graded dietary inclusion of GM, with or without β-mannanase supplementation, on growth performance and nutrient utilization in broiler chickens. Overall, the results demonstrate that the physiological response to GM is strongly dependent on both inclusion level and bird age, and that β-mannanase supplementation can effectively mitigate, but not fully eliminate, the negative effects associated with higher GM inclusion. This interaction highlights the importance of aligning feed formulation strategies with the developmental stage of the gastrointestinal tract. The observed responses further indicate that the effectiveness of β-mannanase depended on dietary GM inclusion level, supporting the existence of a biologically relevant GM × β-mannanase interaction.

During the starter phase, broilers fed 4% GM supplemented with β-mannanase achieved body weight gain comparable to that of the control group and higher than that observed in birds receiving 8% or 12% GM, indicating that young birds have a limited capacity to tolerate elevated levels of GM. At early ages, the digestive system is still undergoing structural and functional maturation, characterized by incomplete enzyme secretion, reduced absorptive surface area, and limited capacity to cope with viscous digesta. Under these conditions, the residual β-mannans and galactomannans present in GM can markedly increase intestinal viscosity, slowing digesta passage rate, impairing nutrient diffusion, and disrupting normal digestive processes. This interpretation is consistent with previous reports showing depressed growth and feed intake when GM inclusion exceeds 8% in starter diets (Nasrala et al. 2015; Hafeez et al. 2025), as well as with earlier studies describing reduced performance at high GM levels (Gharaei et al. 2012; Mishra et al. 2013; Abu Hafsa et al. 2015). Recommendations from Lee et al. (2005) and Mohayayee and Karimi (2012), suggesting safe inclusion levels of 5–6% GM when combined with β-mannanase, further support the concept of a narrow tolerance window during early growth.

In the present study, the adverse effects observed at higher GM inclusion levels are primarily attributable to non-starch polysaccharides, particularly β-mannans and galactomannans, which increase digesta viscosity and interfere with nutrient utilization (Lee et al. 2003b; Gutierrez et al. 2007). In addition, guar meal contains other anti-nutritional factors, including trypsin inhibitors and tannins, whose concentrations can vary widely depending on genotype and environmental growing conditions (Sabahelkhier et al. 2012). In the present study, the trypsin inhibitor content of GM was comparable to that of soybean meal, suggesting that viscosity-related mechanisms, rather than protease inhibition per se, played a dominant role in limiting performance. This variability in chemical composition may also help explain discrepancies among studies regarding the nutritional value of GM and underscores the importance of considering raw material quality when interpreting experimental outcomes.

In the present study, β-mannanase supplementation substantially improved growth performance and feed efficiency across both feeding phases, confirming its ability to counteract the anti-nutritional effects associated with GM (Milczarek et al. 2023). The enzyme likely exerted its beneficial effects by hydrolyzing β-mannans into smaller oligosaccharides, thereby reducing digesta viscosity and releasing nutrients previously entrapped within the fibrous matrix (Yaqoob et al. 2022). This mechanism enhances nutrient accessibility to endogenous digestive enzymes and improves overall nutrient absorption, as previously described by Lee et al. ([Bibr CR23][Bibr CR24]). In the present study, β-mannanase supplementation substantially improved performance at 8% GM during the starter phase, although complete recovery to control values was not always achieved. However, at 12% GM, even β-mannanase supplementation could only partially alleviate performance losses. This finding suggests a potential enzyme-substrate imbalance, whereby the concentration of β-mannans and other anti-nutritional compounds exceeded the enzyme’s capacity to fully hydrolyze the available substrate. Consequently, viscosity-related constraints on nutrient digestion and absorption likely persisted despite supplementation.

A distinct response pattern emerged in the present study during the grower phase, when birds fed 8% GM achieved higher body weight gain than those receiving either 4% or 12% GM. This shift suggests that broilers develop an increased tolerance to dietary fiber as they age, likely due to enhanced endogenous enzyme secretion, greater intestinal absorptive capacity, and improved regulation of digesta flow. Similar age-related adaptations have been described by Scott and Boldaji (1997), who reported improved utilization of fibrous ingredients in older birds. Nonetheless, the persistent reduction in performance observed at 12% GM, even during the grower phase, suggests that a critical viscosity threshold may have been reached, beyond which nutrient utilization becomes increasingly compromised and cannot be fully restored by enzymatic supplementation.

These age-related differences in growth performance were closely reflected in nutrient utilization responses. Patterns of nutrient utilization closely mirrored growth performance outcomes. Crude protein digestibility declined progressively with increasing GM inclusion in both phases, reflecting the combined effects of increased digesta viscosity, reduced enzyme–substrate interaction, and possible binding of dietary protein within fibrous cell wall structures (Lv et al. 2013). β-mannanase supplementation significantly improved protein digestibility, particularly at moderate GM levels, supporting the hypothesis that enzymatic degradation of β-mannans reduces nutrient encapsulation and enhances protein availability (Dawood and Shi 2022).

AME remained unchanged at 4% GM but declined significantly at 8% and 12% GM, suggesting that energy utilization is particularly sensitive to the physicochemical properties of GM and to changes in digesta viscosity. The limited recovery of AME at the highest inclusion level further indicates that enzyme supplementation alone may be insufficient to fully overcome the energetic penalties associated with excessive fiber intake.

Beyond the immediate nutritional and performance implications, the findings of this study have broader relevance for poultry production systems worldwide. Guar is a drought-tolerant legume cultivated not only in Pakistan and India but also in other semi-arid and arid regions facing increasing pressure on water and land resources (Gautam et al. 2024). The valorization of guar meal, a protein-rich by-product of guar gum extraction, aligns with circular economy principles by converting industrial residues into valuable feed ingredients (Maqsood et al. 2025). In regions characterized by high dependency on imported soybean meal, volatile feed markets, or limited access to conventional protein sources, GM represents a locally available alternative that can enhance feed system resilience.

When strategically incorporated into broiler diets and supported by targeted β-mannanase supplementation, GM can contribute to more sustainable and cost-effective poultry production while maintaining animal performance when inclusion levels are appropriately matched to the birds’ physiological stage. Although the present study demonstrated clear treatment effects under controlled experimental conditions, further research involving larger populations and commercial production systems would be valuable to validate these findings under practical field conditions.

The phase-specific feeding strategy identified in this study (4% GM with β-mannanase during the starter phase and 8% GM with β-mannanase during the grower phase) offers a practical framework that can be adapted across diverse production contexts. This approach allows nutritionists and feed manufacturers to balance economic efficiency, ingredient availability, and physiological suitability, particularly in low- and middle-income regions as well as in areas vulnerable to feed supply chain disruptions.

## Conclusions

This study demonstrated that the successful use of GM in broiler diets depends on both inclusion level and bird age, and can be further supported by β-mannanase supplementation. During the starter phase, 4% GM supplemented with β-mannanase supported growth and nutrient utilization comparable to the control diet, whereas higher inclusion levels negatively affected productive responses. In contrast, broilers in the grower phase tolerated up to 8% GM, with β-mannanase partially mitigating the adverse effects associated with higher inclusion levels. The enzyme improved crude protein digestibility and contributed to better energy utilization, particularly at moderate GM inclusion levels.

These findings provide practical guidance for phase-specific incorporation of GM into broiler feeding programs and support the use of this agro-industrial by-product as an alternative protein source. The adoption of GM, combined with targeted enzyme supplementation, may contribute to reducing dependence on conventional feed ingredients while improving the sustainability and resilience of poultry production systems, particularly in regions with similar agro-climatic conditions and feed resource constraints. Future studies should focus on validating these phase-specific feeding strategies under commercial production conditions and evaluating their long-term effects on carcass quality, bird health, economic efficiency, and environmental sustainability.

## Data Availability

The datasets generated during and/or analysed during the current study are not publicly available due to reasons of sensitivity but are available from the corresponding author on reasonable request.
